# Excess mortality and life-years lost in people with bipolar disorder: an 11-year population-based cohort study

**DOI:** 10.1017/S2045796021000305

**Published:** 2021-05-28

**Authors:** J. K. N. Chan, C. S. M. Wong, N. C. L. Yung, E. Y. H. Chen, W. C. Chang

**Affiliations:** 1 Department of Psychiatry, Queen Mary Hospital, The University of Hong Kong, Hong Kong, Hong Kong; 2 State Key Laboratory of Brain and Cognitive Sciences, The University of Hong Kong, Hong Kong, Hong Kong

**Keywords:** Bipolar disorder, premature mortality, excess life-years lost, mortality trend

## Abstract

**Aims:**

Bipolar disorder is associated with premature mortality, but evidence is mostly derived from Western countries. There has been no research evaluating shortened lifespan in bipolar disorder using life-years lost (LYLs), which is a recently developed mortality metric taking into account illness onset for life expectancy estimation. The current study aimed to examine the extent of premature mortality in bipolar disorder patients relative to the general population in Hong Kong (HK) in terms of standardised mortality ratio (SMR) and excess LYLs, and changes of mortality rate over time.

**Methods:**

This population-based cohort study investigated excess mortality in 12 556 bipolar disorder patients between 2008 and 2018, by estimating all-cause and cause-specific SMRs, and LYLs. Trends in annual SMRs over the 11-year study period were assessed. Study data were retrieved from a territory-wide medical-record database of HK public healthcare services.

**Results:**

Patients had higher all-cause [SMR: 2.60 (95% CI: 2.45–2.76)], natural-cause [SMR: 1.90 (95% CI: 1.76–2.05)] and unnatural-cause [SMR: 8.63 (95% CI: 7.34–10.03)] mortality rates than the general population. Respiratory diseases, cardiovascular diseases and cancers accounted for the majority of deaths. Men and women with bipolar disorder had 6.78 (95% CI: 6.00–7.84) years and 7.35 (95% CI: 6.75–8.06) years of excess LYLs, respectively. The overall mortality gap remained similar over time, albeit slightly improved in men with bipolar disorder.

**Conclusions:**

Bipolar disorder is associated with increased premature mortality and substantially reduced lifespan in a predominantly Chinese population, with excess deaths mainly attributed to natural causes. Persistent mortality gap underscores an urgent need for targeted interventions to improve physical health of patients with bipolar disorder.

## Introduction

Bipolar disorder is a severe mental disorder which affects more than 1% of the population and constitutes one of the main causes of disability worldwide (McIntyre *et al*., 2020). People with bipolar disorder have markedly elevated risk of premature mortality (Hayes *et al*., 2015), with approximately 8–15-years shorter lifespan relative to the general population (Chang *et al*., 2011; Laursen, 2011; Kodesh *et al*., 2012; Ajetunmobi *et al*., 2013; Crump *et al*., 2013; Kessing *et al*., 2015; Pan *et al*., 2020). Excess deaths associated with bipolar disorder are mainly attributed to natural causes, particularly cardiovascular diseases, respiratory diseases and cancers (Crump *et al*., 2013; Hayes *et al*., 2015). Accumulating evidence indicates that such mortality gap has persisted (Ajetunmobi *et al*., 2013; Crump *et al*., 2013) or widened (Hayes *et al*., 2017; Pan *et al*., 2017; Hansen *et al*., 2019; Lomholt *et al*., 2019) in recent decades despite overall improvement in life expectancy in the general population due to enhanced healthcare. Physical health inequalities experienced by people with bipolar disorder thus represent a serious public health challenge that warrants urgent attention. Comprehensive evaluation of premature mortality patterns associated with bipolar disorder is crucial for developing effective strategies and optimising healthcare service delivery to reduce avoidable deaths in this vulnerable population.

Notably, literature on excess mortality in bipolar disorder were mostly derived from Western countries (Hayes *et al*., 2015) and their findings may not be generalisable to other areas owing to substantial cross-regional variation in healthcare systems, sociocultural context and population health indices. Until now, very few studies have been conducted in Asia in this respect (Saku *et al*., 1995; Pan *et al*., 2017, 2020) and were limited by small sample size and short follow-up duration. In general, previous research was hampered by several important methodological constraints including sampling patients with psychiatric inpatient treatment only with subsequent bias towards greater illness severity (Saku *et al*., 1995; Ösby *et al*., 2001; Ajetunmobi *et al*., 2013; Hoang *et al*., 2013; Laursen *et al*., 2013), lack of evaluation for cause-specific mortality (Chang *et al*., 2010; Kodesh *et al*., 2012; Ajetunmobi *et al*., 2013; Medici *et al*., 2015; Pan *et al*., 2017; Hansen *et al*., 2019; Lomholt *et al*., 2019) or focus only on one specific natural cause of death for analysis (Saku *et al*., 1995; Laursen *et al*., 2013; Westman *et al*., 2013; Hayes *et al*., 2017). Alternatively, a growing number of studies have assessed the impact of premature mortality on survival in bipolar disorder using years of life lost (YLLs), which are based on estimating life expectancy at a single fixed age (mostly at birth or 15 years). This approach, however, does not reflect the underlying age-of-onset distribution of the disorder and implies an assumption that the estimated life expectancy is interpreted as the expected lifespan for patients who have had the disorder since the set fixed age (Plana-Ripoll *et al*., 2020). In fact, recent Danish register-based studies which investigated the longevity gap between people with mental disorders and the general population using life-years lost (LYLs; Erlangsen *et al*., 2017; Plana-Ripoll *et al*., 2019), a novel and more realistic measure taking into consideration different ages of onset of the disorder (Andersen, 2017), revealed that past estimates of reduced life expectancy tend to overestimate mortality difference. Thus far, there has been no study examining LYLs for bipolar disorder.

In this large population-based cohort study, we aimed to comprehensively examine the risk of premature mortality associated with bipolar disorder over 11 years in Hong Kong (HK), a metropolitan city located at the southeastern tip of China with a population of approximately 7.5 million, utilising data retrieved from a territory-wide medical-record database of public healthcare services. Specifically, we adopted two mortality metrics, namely SMR (for all-cause and cause-specific mortality) and LYLs to quantify the magnitude of excess mortality among patients with bipolar disorder compared with the general population. Changes in SMRs across the study period were also assessed to clarify whether the mortality gap improved or worsened over time.

## Methods

### Data source

Population statistics and information on all registered deaths in HK between 2008 and 2018 (each calendar year from 2008 to 2018, inclusive) were obtained from the Census and Statistics Department. Data of the patient cohort were extracted from the Clinical Data Analysis and Reporting System (CDARS; Hospital Authority, 2003), a territory-wide electronic health-record database developed by the Hospital Authority (HA) which is a statutory body delivering government-subsidised, universal health coverage to all HK residents (approximately 92% being Chinese) by managing all public hospitals, specialist and general outpatient clinics in HK. Detailed description of CDARS has been reported elsewhere (Cheung *et al*., 2007). Briefly, CDARS is an integrated, longitudinal patient electronic record system capturing clinical data across all healthcare settings of HA facilities. The database contains patients’ demographics and clinical information including diagnoses, attendances to outpatient clinics and emergency departments and hospital admissions. Data on dates and causes of death were retrieved from CDARS via internal linkage to regional death registries from the Immigration Department. Clinical data are collected and entered into computerised clinical-management system (CMS) by treating clinicians and other healthcare professionals, and are then transferred to CDARS for audit and research purposes. CDARS generates unique, anonymised patient identifiers to protect privacy and to link all medical records. This database has been used to conduct high-quality population-based studies on various psychiatric disorders including schizophrenia and other psychoses (Chang *et al*., 2020; Yung *et al*., 2020, 2021; Chan *et al*., 2021).

### Study population

The study period was between 1 January 2008 and 31 December 2018. We identified all individuals who were diagnosed with bipolar disorder for public psychiatric inpatient admissions or outpatient care between 1 January 2002 and 31 December 2018 (computerised CMS for psychiatric services implemented since 1 January 2000), and aged ⩾15 years during the study period as the study population. Diagnosis of bipolar disorder was recorded and verified by the International Classification of Diseases, 10th revision (ICD10 codes: F30 and F31). Final diagnostic ascertainment took into consideration the longitudinal illness course (Chang *et al*., 2009), and those identified patients with subsequent diagnostic change to schizophrenia or schizoaffective disorder (as their most-recently assigned principal diagnosis) before the end of follow-up were excluded. Follow-up of the patient cohort began on the date of first-recorded diagnosis of bipolar disorder. For patients who had been assigned with a diagnosis of bipolar disorder before the study period, their follow-up start-date was defined as 1 January 2008. The cohort was followed forward until the date of death or 31 December 2018, whichever came first. The study was approved by the Institutional Review Board of the University of Hong Kong/Hospital Authority Hong Kong West Cluster. The study data were anonymised and individual patient records were completely unidentifiable during the analysis. Since our study was based on medical-record database, the requirement for informed consent was waived.

### Study outcomes

Causes of death were classified according to ICD10 codes (online Supplementary Table S1), and were divided into natural and unnatural causes. Natural causes were categorised into infectious and parasitic diseases (A00–B99), neoplasms (C00–D48), cardiovascular diseases (I00–I99), respiratory diseases (J00–J99), digestive diseases (K00–K93) and genitourinary diseases (N00–N99). An array of specific natural causes was also identified for analyses. As data on specific unnatural causes (V01–Y98) were not available, we treated unnatural deaths as a single category for analyses.

### Statistical analysis

Standardised mortality ratios (SMRs) for all-cause and cause-specific deaths were calculated as the primary mortality measures to quantify the relative mortality rate between patients with bipolar disorder and the general population. First, number of observed deaths and person-years of follow-up were computed for each calendar year (2008–2018), sex and age category (by 5-year age bands from 15 to 84 years, and ⩾85 years) for the patient group. The number of person-years for each stratum was multiplied by the corresponding mortality rate in the general population to produce expected number of deaths, indirectly standardising overall mortality ratio by age, sex and calendar year. SMRs were estimated by dividing the observed number of deaths by the expected number of deaths. Crude mortality rates (CMRs) per 100 000 person-years as well as SMRs for all-cause and cause-specific deaths of the patient group over the whole study period, stratified by sex and four broader age groups (15–34, 35–49, 50–64 and ⩾65 years), were then calculated, with 95% confidence intervals (CIs) of SMRs being derived by mid-*P* exact tests. To assess trends in all-cause, natural-cause and unnatural-cause SMRs over time, joinpoint regression analysis was performed which estimated the optimal number of linear slopes and joinpoints with modified Bayesian information criteria (Kim *et al*., 2000). The models incorporated estimated variation for each data point using the standard error of SMRs, and calculated annual percentage change (APC) in SMRs with 95% CIs.

Excess life-years lost (LYLs) for all-cause mortality were also calculated as a complementary mortality measure. Following the method adopted in previous research for LYL estimation (Andersen, 2017; Erlangsen *et al*., 2017; Plana-Ripoll *et al*., 2019; Yung *et al*., 2021), we first computed average life expectancy at first-recorded diagnosis of bipolar disorder (as a proxy for age of onset), which takes into account varying ages at illness onset. Life expectancies of patients were calculated for every age at diagnosis from 15 years until a set upper-age limit (95 years in the current study). These were then combined into average life expectancy until age 95 years, weighted by the number of patients at each of the particular ages at diagnosis (Erlangsen *et al*., 2017; Plana-Ripoll *et al*., 2019). LYLs of patients referred to the average number of years lost due to death and was computed by subtracting the average life expectancy from 95 years (i.e. remaining 95-year restricted life expectancy). Similarly, in general population, LYLs denoted the average number of years lost before 95 years for a group of people of the same sex who were alive at ages corresponding to the age-of-onset distribution of those patients with bipolar disorder. Excess LYLs for men and women with bipolar disorder were then calculated as the difference in LYLs between patients and the general population. Thus, excess LYLs referred to the average number of years that patients lost in excess of that observed in the general population of the same sex and age (Andersen, 2017; Erlangsen *et al*., 2017). The 95% CIs of excess LYLs were derived from non-parametric bootstrap with 500 iterations. We also decomposed total LYLs for patients into loss attributable to specific causes of death based on a completing risk model to generate cause-specific LYLs (Andersen, 2013). This enables clarification of how specific death causes contributing to premature mortality in bipolar disorder. Excess LYLs were estimated using the R package *lillies* (Plana-Ripoll *et al*., 2020).

## Results

The study population included 12 556 patients with bipolar disorder (men: 4928; women: 7628) with 106 147 persons-years of follow-up (men: 40 739; women: 65 408), and a total of 1042 deaths, of which 821 (78.8%) had a known cause. The numbers of person-years and cause-specific death counts for each demographic subgroup of patients are listed in online Supplementary Table S2.

### All-cause mortality rates and excess LYLs

All-cause SMR for bipolar disorder was significantly increased in the total sample (SMR = 2.60, 95% CI: 2.45–2.76) and in each demographic subgroup relative to the general population (Tables 1 and 2). All-cause SMR for women and for men was 2.82 (95% CI: 2.59–3.06) and 2.38 (95% CI: 2.18–2.60), respectively. It decreased with increasing age and was particularly high in the youngest age-group (SMR = 10.18, 95% CI: 7.94–12.71). Bipolar disorder was associated with shorter remaining life expectancy relative to the general population. Estimated LYLs for men with bipolar disorder was 19.90 years compared with 13.12 years for men in the general population, with a difference of 6.78 (95% CI 6.00–7.84) excess LYLs. Women with bipolar disorder and in the general population had LYLs of 15.86 and 8.51 years, respectively, with a difference of 7.35 (95% CI 6.75–8.06) excess LYLs.

**Table 1.** Crude mortality rates, all-cause and cause-specific standardised mortality ratios of patients with bipolar disorder

	Total		Men		Women	
Cause of death	CMR^a^	SMR (95% CI)	CMR	SMR (95% CI)	CMR	SMR (95% CI)
All causes	1119.2	2.60 (2.45–2.76)	1354.8	2.38 (2.18–2.60)	972.0	2.82 (2.59–3.06)
Natural causes	712.1	1.90 (1.76–2.05)	840.8	1.70 (1.51–1.89)	631.7	2.12 (1.90–2.34)
Cardiovascular diseases	123.5	1.33 (1.10–1.59)	150.8	1.23 (0.92–1.58)	106.5	1.44 (1.10–1.83)
Ischaemic heart disease	50.5	1.33 (0.98–1.74)	81.0	1.42 (0.95–1.99)	31.4	1.21 (0.72–1.84)
Non-ischaemic heart diseases	29.0	1.56 (1.03–2.21)	19.6	0.95 (0.38–1.79)	34.9	2.01 (1.23–2.99)
Cerebrovascular diseases	38.7	1.30 (0.91–1.76)	41.9	1.15 (0.64–1.80)	36.7	1.44 (0.89–2.12)
Neoplasms	169.7	1.03 (0.87–1.20)	173.2	0.81 (0.62–1.02)	167.5	1.25 (1.01–1.51)
Lung cancer	31.2	0.73 (0.49–1.03)	47.5	0.75 (0.43–1.14)	20.9	0.72 (0.37–1.18)
Liver cancer	10.7	0.61 (0.29–1.05)	11.2	0.35 (0.09–0.78)	10.5	1.19 (0.43–2.34)
Colon cancer	32.2	2.14 (1.44–2.97)	30.7	1.67 (0.83–2.80)	33.2	2.56 (1.54–3.84)
Breast cancer	18.3	1.58 (0.92–2.42)	0	0	29.7	1.59 (0.92–2.43)
Respiratory diseases	295.4	4.09 (3.63–4.59)	268.7	3.59 (3.00–4.23)	249.6	4.70 (3.97–5.51)
Non-aspiration pneumonia	247.0	4.72 (4.13–5.35)	307.3	4.46 (3.66–5.33)	209.4	5.00 (4.14–5.93)
Aspiration pneumonia	18.3	8.04 (4.67–12.32)	19.6	6.90 (2.74–12.96)	17.5	9.10 (4.33–15.61)
Chronic obstructive pulmonary disease	7.5	0.69 (0.27–1.30)	13.9	0.63 (0.20–1.30)	3.5	0.95 (0.09–2.71)
Digestive diseases	37.6	2.59 (1.80–3.52)	50.3	2.51 (1.48–3.80)	29.7	2.69 (1.56–4.12)
Liver diseases	10.7	2.06 (0.98–3.53)	16.8	1.96 (0.71–3.85)	7.0	2.21 (0.57–4.90)
Pancreaticobiliary diseases	6.4	2.87 (1.03–5.63)	2.8	1.12 (0.01–4.38)	8.7	4.19 (1.32–8.66)
Genitourinary diseases	42.9	2.30 (1.64–3.07)	44.7	2.18 (1.24–3.37)	41.9	2.39 (1.53–3.44)
Renal failure	22.6	1.63 (1.01–2.40)	30.7	1.93 (0.96–3.24)	17.5	1.39 (0.66–2.39)
Infectious and parasitic diseases	42.9	3.78 (2.70–5.04)	53.1	3.46 (2.08–5.19)	36.7	4.12 (2.55–6.07)
Unnatural causes	169.7	8.63 (7.34–10.03)	192.7	6.55 (5.09–8.18)	155.3	11.46 (9.20–13.96)

CI, confidence interval; CMR, crude mortality rate; SMR, standardised mortality ratio (standardised for age, sex and calendar year).

a CMRs are presented as the numbers of deaths per 100 000 person-years.

**Table 2.** Crude mortality rates, all-cause and cause-specific standardised mortality ratios of patients with bipolar disorder by age group

	15–34 years		35–49 years		50–64 years		⩾65 years	
Cause of death	CMR^a^	SMR (95% CI)	CMR	SMR (95% CI)	CMR	SMR (95% CI)	CMR	SMR (95% CI)
All causes	338.5	10.18 (7.94–12.71)	433.7	4.27 (3.60–5.00)	1072.0	2.98 (2.66–3.32)	4767.6	2.03 (1.86–2.21)
Natural causes	33.9	2.41 (0.95–4.52)	161.9	2.11 (1.58–2.72)	678.7	2.13 (1.85–2.44)	3726.4	1.78 (1.61–1.96)
Cardiovascular diseases	4.8	1.55 (0.01–6.10)	27.5	1.57 (0.71–2.76)	128.8	2.04 (1.43–2.74)	621.1	1.10 (0.86–1.38)
Ischaemic heart disease	0	0	3.1	0.44 (0.01–1.73)	62.7	2.22 (1.32–3.37)	255.7	1.13 (0.75–1.59)
Non-ischaemic heart diseases	0	0	9.2	2.68 (0.51–6.57)	31.3	2.89 (1.31–5.09)	137.0	1.17 (0.65–1.84)
Cerebrovascular diseases	1.8	1.48 (0.07–7.30)	8.4	1.31 (0.71–2.23)	44.9	1.98 (1.54–2.51)	21.0	0.94 (0.79–1.10)
Neoplasms	14.5	1.91 (0.36–4.67)	55.0	1.15 (0.68–1.74)	233.2	1.16 (0.90–1.45)	639.3	0.89 (0.69–1.11)
Lung cancer	0	0	3.1	0.38 (0.01–1.48)	45.3	0.92 (0.49–1.49)	137.0	0.66 (0.37–1.04)
Liver cancer	0	0	9.2	1.96 (0.37–4.81)	10.4	0.45 (0.09–1.11)	36.5	0.49 (0.13–1.09)
Colon cancer	0	0	3.1	0.99 (0.01–3.89)	41.8	2.62 (1.35–4.32)	155.3	2.04 (1.18–3.12)
Breast cancer	0	0	15.3	2.16 (0.68–4.47)	34.8	1.76 (0.84–3.02)	18.3	0.76 (0.07–2.17)
Respiratory diseases	9.7	5.38 (0.51–15.43)	48.9	11.04 (6.29–17.11)	191.4	7.62 (5.74–9.76)	1844.9	3.48 (3.01–3.97)
Non-aspiration pneumonia	0	0	33.6	11.34 (5.6–19.03)	160.1	9.70 (7.10–12.70)	1580.1	4.05 (3.47–4.68)
Aspiration pneumonia	4.8	43.13 (0.02–169.08)	3.1	22.66 (0.01–88.83)	17.4	21.99 (6.94–45.50)	91.3	5.50 (2.62–9.43)
COPD	0	0	3.1	15.87 (0.01–62.21)	7.0	2.16 (0.20–6.18)	36.5	0.44 (0.11–0.98)
Digestive diseases	0	0	12.2	4.37 (1.14–9.71)	38.3	3.30 (1.64–5.54)	182.7	2.19 (1.33–3.25)
Liver diseases	0	0	9.2	5.68 (1.07–13.93)	17.4	2.87 (0.91–5.93)	18.3	0.78 (0.07–2.24)
Pancreaticobiliary diseases	0	0	0	0	3.5	3.73 (0.01–14.63)	45.7	2.88 (0.91–5.97)
Genitourinary diseases	0	0	12.2	7.70 (2.00–17.10)	27.8	3.07 (1.31–5.57)	255.7	1.97 (1.31–2.77)
Renal failure	0	0	12.2	10.48 (2.73–23.26)	13.9	1.95 (0.51–4.32)	118.7	1.25 (0.66–2.02)
Infectious and parasitic diseases	4.8	6.93 (0.01–27.17)	6.1	2.47 (0.23–7.08)	59.2	7.61 (4.42–11.66)	182.7	2.70 (1.65–4.02)
Unnatural causes	222.4	16.58 (12.14–21.72)	125.2	8.47 (6.07–11.26)	160.1	8.67 (6.35–11.36)	228.3	4.64 (3.00–6.64)

CI, confidence interval; CMR, crude mortality rate; COPD, chronic obstructive pulmonary disease, SMR, standardised mortality ratio (standardised for age, sex and calendar year).

a CMRs are presented as the numbers of deaths per 100 000 person-years.

### Natural-cause and unnatural-cause mortality rates

SMRs for natural and unnatural causes were significantly increased in the total sample (natural-cause SMR = 1.90, 95% CI: 1.76–2.05; unnatural-cause SMR = 8.63, 95% CI: 7.34–10.03) and in each demographic subgroup of patients with bipolar disorder, except the youngest age-group who showed no significant increase in natural-cause SMR (Tables 1 and 2). Both natural-cause and unnatural-cause SMRs were higher for women than for men, and generally decreased with age, with markedly elevated unnatural-cause SMR in the youngest age-group (16.58, 95% CI: 12.14–21.72).

Natural causes accounted for most of the known-cause deaths in patients. Approximately two-thirds of all known-cause deaths were attributed to respiratory diseases, cancers and cardiovascular diseases (online Supplementary Table S2). Respiratory diseases represented the leading cause of death and accounted for around one-thirds of all known-cause deaths. Cancers and cardiovascular diseases contributed to about 1 in 5 and 1 in 7 known-cause deaths, respectively. Unnatural causes accounted for approximately one-fifths of known-cause deaths. Men had generally higher CMRs for most listed causes of death than women (Table 1). Mortality rate for most natural causes increased with age, while that for unnatural causes was comparatively higher in the youngest and the oldest age-groups (Table 2). Analyses on cause-specific LYLs for bipolar disorder generally revealed consistent results. Respiratory diseases accounted for the largest share of LYLs for patients of both sexes and the degree of contribution increased with age, particularly in men. Cancers and cardiovascular diseases also constituted a major share to LYLs due to natural-cause deaths for both men and women with bipolar disorder, with the proportion of their contributions mildly increased with age. By contrast, unnatural causes accounted for a decreasing share of LYLs with age in patients of both sexes (online Supplementary Fig. S3 for detailed results).

As shown in Table 1, respiratory diseases displayed the highest SMR among natural-cause categories (4.09, 95% CI: 3.63–4.59), with pneumonia (non-aspiration and aspiration) showing the highest SMRs across all listed specific natural causes. Conversely, SMR for chronic obstructive pulmonary disease (COPD) was not elevated. We observed increased SMR for cardiovascular disease (1.33, 95% CI: 1.10–1.59), with subgroup analyses revealing significantly elevated relative mortality rate among women and individuals aged 50–64 years. Women, but not men or the total sample, with bipolar disorder had elevated SMR for cancers (1.25, 95% CI: 1.01–1.51). Among various cancer types, SMR was only significantly increased for colon cancer, specifically among women and age-groups ⩾50 years (Table 1 and 2). Bipolar disorder was also associated with significantly increased SMRs for infectious and parasitic diseases, digestive diseases and genitourinary diseases.

### Trends in mortality rates over study period

**Fig. 1.**
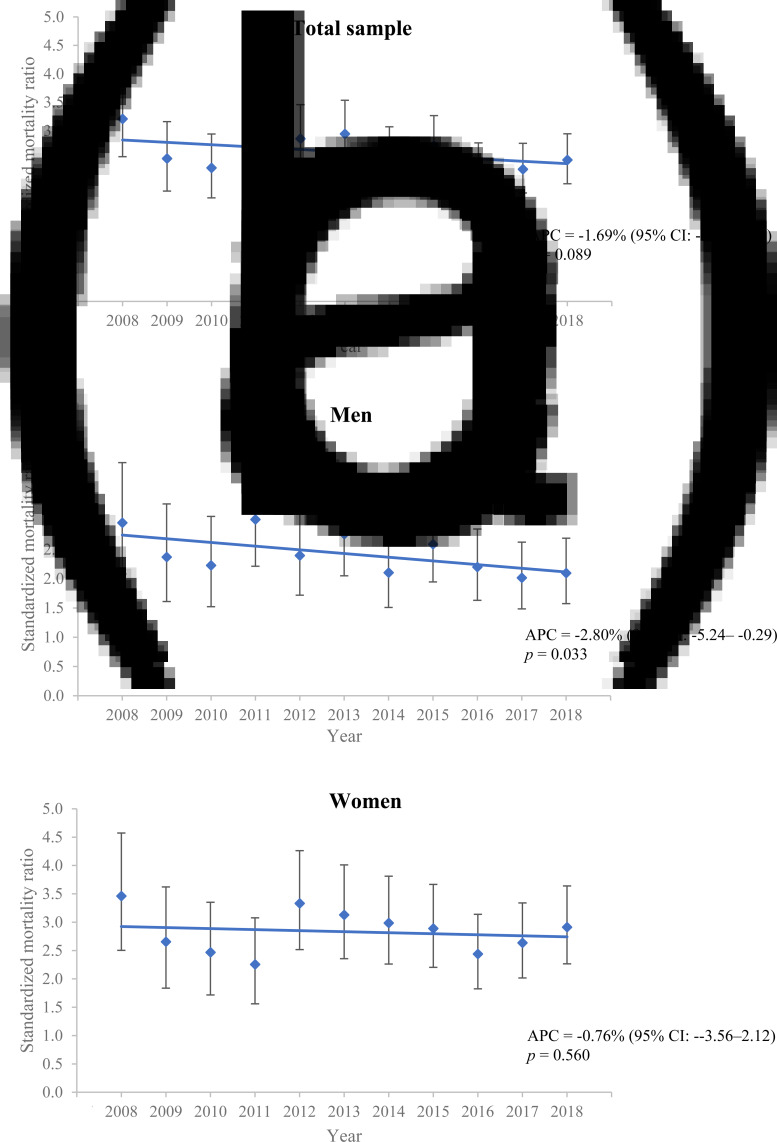
Annual all-cause standardised mortality ratios of patients with bipolar disorder over study period: (a) total sample, (b) men and (c) women. *Note*: APC, annual percentage change; CI, confidence interval.

Joinpoint regression fitted a linear model of all-cause, natural-cause and unnatural-cause SMRs with no joinpoints over time for the total sample, men and women subgroups. As shown in Fig. 1, both the total sample and women exhibited a stable trend of all-cause SMRs over a period of 2008–2018. A trend in all-cause SMRs in men with bipolar disorder indicated a small degree of reduction over study period, with a significant APC in rate of −2.80% (95% CI: −5.24 to −0.29). There was no significant APC in natural-cause and unnatural-cause SMRs over time for the total sample or in either sex (Table 3, Supplementary Figs S1 and S2).

**Table 3.** Annual percentage change in all-cause, natural-cause and unnatural-cause standardised mortality ratios of patients with bipolar disorder from 2008 to 2018^a^

	APC	95% CI	*P* value
All-cause SMR			
Total sample	−1.69	−3.67 to 0.32	0.089
Men	−2.80	−5.24 to −0.29	0.033
Women	−0.76	−3.56 to 2.12	0.560
Natural-cause SMR			
Total sample	−1.00	−4.46 to 2.58	0.538
Men	−2.40	−6.66 to 2.05	0.249
Women	0.14	−3.91 to 4.36	0.940
Unnatural-cause SMR			
Total sample	−3.24	−9.09 to 3.00	0.263
Men	−4.57	−13.43 to 5.20	0.306
Women	−0.47	−8.20 to 7.91	0.898

APC, annual percentage change; CI, confidence interval; SMR, standardised mortality ratio.

a Joinpoint regression analyses were performed to calculate annual percentage change of SMRs with 95% CIs over the study period.

## Discussion

To our knowledge, this is the first study using LYLs complementary with relative mortality risk measure (SMR) to evaluate the excess mortality associated with bipolar disorder. Our results showed that patients with bipolar disorder had 2.6-fold increased mortality risk compared with the general population. Both men and women with bipolar disorder exhibited a substantially shorter lifespan than the general population, with approximately 7 years of excess LYLs. Natural causes, specifically respiratory diseases, cardiovascular diseases and cancers, accounted for the majority of known-cause deaths, while unnatural cause had markedly elevated SMR, particularly among patients aged <35 years. Generally, the mortality gap persisted over 11 years, albeit slightly improved in men with bipolar disorder.

Our finding on all-cause SMR for bipolar disorder is broadly consistent with the literature which reported 2–3 times greater overall mortality risk than the general population (Chang *et al*., 2010; Crump *et al*., 2013; Laursen *et al*., 2013; Westman *et al*., 2013), and is in line with a recent meta-analysis reporting all-cause summary SMR of 2.05 for bipolar disorder (Hayes *et al*., 2015). In particular, our results are similar to the findings of Taiwan studies (the only recently published Asian studies in this respect), which revealed that all-cause SMR for bipolar disorder ranged from 2.39 to 2.65 (Pan *et al*., 2017, 2020). Of note, although we affirmed that bipolar disorder was associated with considerably shortened life expectancy after diagnosis, our observed magnitude of longevity gap is smaller than most previous estimates based on YLLs, ranging between 10 and 15 years shorter in lifespan than the general population (Chang *et al*., 2011; Ajetunmobi *et al*., 2013; Laursen *et al*., 2013; Pan *et al*., 2020). Recent research, however, indicates that LYL method yields more precise, though more conservative, estimates than prior measures of reduced life expectancy by incorporating variations in age of onset of the disorder into life expectancy calculation (Erlangsen *et al*., 2017; Plana-Ripoll *et al*., 2019). This also concurs with the results of a Danish study which demonstrated that difference in remaining life expectancy between bipolar disorder patients and the general population decreased from 12.0 to 8.7 years for men and 10.6 to 8.3 years for women, when the set age of onset for lifespan estimation increased from 25 to 45 years (Kessing *et al*., 2015). Taken together, adoption of LYLs enables more accurate quantification of life expectancy reduction for bipolar disorder, which in turn facilitates policy planning, resource allocation and service development for improvement in physical health outcomes.

Until now, very few studies have comprehensively examined cause-specific SMRs for natural deaths in bipolar disorder. Consistent with the literature (Crump *et al*., 2013; Hayes *et al*., 2015), we found that cardiovascular diseases, respiratory diseases and cancers represented the major contributors to natural-cause mortality in bipolar disorder. This also accords with the results of our cause-specific LYL analyses demonstrating that these three disease categories accounted for the major share of total LYLs due to natural-cause deaths for patients of both sexes. In fact, evidence has shown that bipolar disorder is associated with an increased incidence of cardiovascular diseases (Correll *et al*., 2017), but inequitable cardiac care including lower receipt of revascularisation interventions (Wu *et al*., 2013; Heiberg *et al*., 2020) and under-prescription of cardioprotective medications (Smith *et al*., 2013; Laursen *et al*., 2014). Our result of SMR for cardiovascular diseases was in the lower end of those previously reported (mostly around 2-fold increased mortality risk; Ösby *et al*., 2001; Crump *et al*., 2013; Laursen *et al*., 2013; Westman *et al*., 2013; Hayes *et al*., 2015). We observed that women, but not men, with bipolar disorder had raised cardiovascular-related SMR. This is contrary to most earlier research which found elevated mortality risk in both sexes (Ösby *et al*., 2001; Crump *et al*., 2013; Westman *et al*., 2013; Pan *et al*., 2020), although some revealed slightly higher cardiovascular mortality in women than in men (Ösby *et al*., 2001; Crump *et al*., 2013). A recent study further demonstrated that female patients with bipolar disorder were more likely to die of undiagnosed cardiovascular diseases than male counterparts (Heiberg *et al*., 2019). Lifestyle modification, early detection and optimal treatment of comorbid diabetes, dyslipidaemia and hypertension should be implemented to reduce cardiovascular mortality in bipolar disorder. Our findings that respiratory diseases had the highest SMR across all natural-cause categories accord with past studies indicating respiratory diseases as one of the leading causes of death for bipolar disorder with high relative mortality rate (Ösby *et al*., 2001; Crump *et al*., 2013; Hayes *et al*., 2015). Pneumonia accounted for the majority of our observed respiratory-related deaths for bipolar disorder. This aligns with evidence showing that bipolar disorder patients display higher risk of pneumonia and poorer post-pneumonia outcomes than the general population (Schoepf and Heun, 2014; Davydow *et al*., 2016; Li *et al*., 2018). However, we noted that the proportion of respiratory-related deaths attributed to COPD was small and SMR for COPD was not elevated in bipolar disorder. This is at odds with one previous study demonstrating increased COPD-related mortality risk in bipolar disorder patients (Crump *et al*., 2013). One possible explanation is that a significant proportion of our patients with pneumonia as their assigned death cause might have underlying COPD, a risk factor of and one of the most frequent comorbidities with pneumonia which is a major cause of death among COPD patients. As our mortality analysis was based on a single-cause of death per deceased person, this may introduce inaccuracy in death-cause assignment for patients with multi-morbidity, which is nonetheless more common in bipolar disorder than the general population. Alternatively, our finding of non-elevated mortality rate for cancers in the overall sample is in contrast to a meta-analysis reporting modestly increased cancer-related SMR for bipolar disorder (Hayes *et al*., 2015). Yet, in agreement with two prior register-based studies (Ösby *et al*., 2001; Crump *et al*., 2013), we found that women (but not men) with bipolar disorder had increased cancer-related SMR (1.25), similar to their reported estimates (ranged: 1.2–1.3). Furthermore, bipolar disorder was associated with significantly elevated SMR for colon cancer only (but not other cancer types), specifically among women. This is consistent with the only previous study which also examined SMRs for specific cancer types in bipolar disorder (Crump *et al*., 2013). It should, however, be noted that our cancer-related SMRs might still be underestimated owing to the missing data on patients’ death cause and potential misclassification bias in cause-of-death ascertainment. Non-elevated cancer-related SMRs might also be attributable to a markedly reduced lifespan in bipolar disorder patients, rendering them more likely to die from non-cancer causes, especially among elderly-age group (Plana-Ripoll *et al*., 2019).

Patients with bipolar disorder had approximately 8 times greater risk of dying from unnatural causes than the general population. This finding is concordant with the unnatural-cause summary SMR of 7.42 reported by a recent meta-analysis (Hayes *et al*., 2015). Such mortality rate was particularly elevated among the youngest-age group, with up to 16-fold increased risk for unnatural-cause deaths. Our cause-specific LYLs analyses also revealed convergent findings that the degree of contribution to the overall LYLs by unnatural-cause deaths was greatest at younger ages, and decreased with age. Notably, our lack of information regarding specific unnatural causes precludes us from clarifying mortality risk of suicide which has been consistently shown to predominate unnatural deaths for bipolar disorder. Nonetheless, our results are in line with past studies indicating that suicide risk is highest during the early course of illness (Ösby *et al*., 2001; Crump *et al*., 2013; Medici *et al*., 2015), which typically emerges in late adolescence and early adulthood. This underscores the importance of early intervention for suicide prevention in bipolar disorder.

Although our analysis suggested small, albeit statistically significant, reduction in annual all-cause mortality rate over time in men with bipolar disorder, the overall mortality gap remained largely unchanged over 11 years. Both natural-cause and unnatural-cause mortality differences also showed no significant improvement across the study period in either sex or in the overall sample. Our results thus agree with most previous studies (Medici *et al*., 2015; Hayes *et al*., 2017; Pan *et al*., 2017, 2020; Hansen *et al*., 2019; Lomholt *et al*., 2019), and suggest that bipolar disorder patients still experience pronounced physical health disparities and have not benefited equally from enhanced medical care with life expectancy improvement as compared to the general population.

Several study limitations should be noted. First, several demographic variables including educational level, socioeconomic or employment status and a number of premature mortality risk factors such as unhealthy lifestyles and obesity were not adequately recorded in medical database and thus were not included in the analysis. Second, missing data on patients’ death causes may compromise the accuracy in evaluating cause-specific SMRs. Third, as information on specific unnatural causes was not available, we were not able to investigate mortality risk for individual categories of unnatural deaths including suicide and accidents. Fourth, the study data did not contain information denoting the disorder subtypes, precluding us from examining potential differences between bipolar I and II disorders in mortality rate and life expectancy. Fifth, patients’ age of illness onset was defined by age of first-recorded diagnosis of bipolar disorder. However, previous studies showed that there could be significant delays between illness onset and ascertainment of bipolar disorder diagnosis (Dagani *et al*., 2017), especially among patients who initially present with depressive episode (Berk *et al*., 2007). Our study may thus overestimate the actual age of illness onset, and then underestimate LYLs for bipolar disorder. Sixth, a proportion of patients may have first-recorded diagnosis before the study period. This would result in survival bias and may contribute to our results of reduced relative mortality risk with age. Seventh, our lack of individual-level mortality data for the general population precludes us from estimating cause-specific excess LYLs for bipolar disorder (Plana-Ripoll *et al*., 2020). Eighth, patients’ data were retrieved from medical-record database of public healthcare services managed by HA, and patients who were under private psychiatric care were not included in the study. However, HA is the predominant provider of psychiatric services to individuals with severe mental disorders in HK, and hence the risk of selection bias or missing treated cases of bipolar disorder was minimised. Lastly, as HK is a highly urbanised, densely populated city and is categorised by the World Bank as a high-income economy (World Bank, 2020), our findings may not be generalisable to mainland China or other Asian regions.

In conclusion, this large population-based study indicates that bipolar disorder patients have increased mortality risk and markedly reduced life expectancy in a predominantly Chinese population, with excess mortality being primarily attributable to natural causes. Our findings of persistent mortality gap across the study period highlights an urgent need for further research to identify its underlying causes and to implement multi-level, targeted interventions to promote physical health of bipolar disorder patients so as to significantly reduce their risk of preventable physical morbidity and premature mortality.

## Data Availability

The data that support the findings of this study are available from the corresponding author upon reasonable request.

## Supplementary material

For supplementary material accompanying this paper visit http://dx.doi.org/10.1017/S2045796021000305. click here to view supplementary material
