# Supplementary material for: Excess mortality and life-years lost in people with bipolar disorder: an 11-year population-based cohort study
Source: Epidemiol Psychiatr Sci. 2021 May 28;30:e39. doi: 10.1017/S2045796021000305 (PMC8193965; doi:10.1017/S2045796021000305)
Supplement: Supplementary file 1 [file S2045796021000305sup001.docx]

**Supplementary Materials**

1. **Table S1.** ICD-10 Codes used to define causes of death
2. **Table S2.** Person-years and observed deaths in patients with bipolar disorder by sex and age group
3. **Fig. S1.** Annual natural-cause standardized mortality ratios of patients with bipolar disorder over study period
4. **Fig. S2.** Annual unnatural-cause standardized mortality ratios of patients with bipolar disorder over study period
5. **Fig. S3.** Remaining life expectancy and cause-specific life years lost for men and women with bipolar disorder

**Table S1.** ICD-10 Codes used to define causes of death

| **Causes of death** | **ICD-10 codes** |
| --- | --- |
| Natural causes | A00–R99 |
| Cardiovascular diseases | I00–I99 |
| Ischemic heart disease | I20– I25 |
| Non-ischemic heart diseases | I00–I09, I11, I13, I26–I51 |
| Cerebrovascular diseases | I60–I69 |
| Neoplasms | C00–D49 |
| Lung cancer | C34 |
| Liver cancer | C22 |
| Colon cancer | C18 |
| Breast cancer | C50 |
| Respiratory diseases | J00–J99 |
| Non-aspiration pneumonia | J15–J18 |
| Aspiration pneumonia | J690 |
| Chronic obstructive pulmonary disease | J40–J44 |
| Digestive diseases | K00–K93 |
| Liver diseases | K70–K77 |
| Pancreaticobiliary diseases | K80–K87 |
| Genitourinary diseases | N00–N99 |
| Renal failure | N17–N19 |
| Infectious and parasitic diseases | A00–B99 |
| Unnatural causes | V01–Y98 |

**Table S2.** Person-years and observed deaths in patients with bipolar disorder by sex and age group

|  | Sex |  | Age (years) | | | |
| --- | --- | --- | --- | --- | --- | --- |
|  | Men | Women | 15-34 | 35-49 | 50-64 | ≥65 |
| Person-years | 198607 | 207576 | 56193 | 142298 | 142461 | 65232 |
| Observed deaths by cause |  |  |  |  |  |  |
| All causes | 485 | 557 | 70 | 142 | 308 | 522 |
| Natural causes | 301 | 362 | 7 | 53 | 195 | 408 |
| Cardiovascular diseases | 54 | 61 | 1 | 9 | 37 | 68 |
| Ischemic heart disease | 29 | 18 | 0 | 1 | 18 | 28 |
| Non-ischemic heart diseases | 7 | 20 | 0 | 3 | 9 | 15 |
| Cerebrovascular diseases | 15 | 21 | 0 | 5 | 10 | 21 |
| Neoplasms | 62 | 96 | 3 | 18 | 67 | 70 |
| Lung cancer | 17 | 12 | 0 | 1 | 13 | 15 |
| Liver cancer | 4 | 6 | 0 | 3 | 3 | 4 |
| Colon cancer | 11 | 19 | 0 | 1 | 12 | 17 |
| Breast cancer | 0 | 17 | 0 | 5 | 10 | 2 |
| Respiratory diseases | 132 | 143 | 2 | 16 | 55 | 202 |
| Non-aspiration pneumonia | 110 | 120 | 0 | 11 | 46 | 173 |
| Aspiration pneumonia | 7 | 10 | 1 | 1 | 5 | 10 |
| Chronic obstructive pulmonary disease | 5 | 2 | 0 | 1 | 2 | 4 |
| Digestive diseases | 18 | 17 | 0 | 4 | 11 | 20 |
| Liver diseases | 6 | 4 | 0 | 3 | 5 | 2 |
| Pancreaticobiliary diseases | 1 | 5 | 0 | 0 | 1 | 5 |
| Genitourinary diseases | 16 | 24 | 0 | 4 | 8 | 28 |
| Renal failure | 11 | 10 | 0 | 4 | 4 | 13 |
| Infectious and parasitic diseases | 19 | 21 | 1 | 2 | 17 | 20 |
| Unnatural causes | 69 | 89 | 46 | 41 | 46 | 25 |
| Unknown causes | 115 | 106 | 17 | 48 | 67 | 89 |


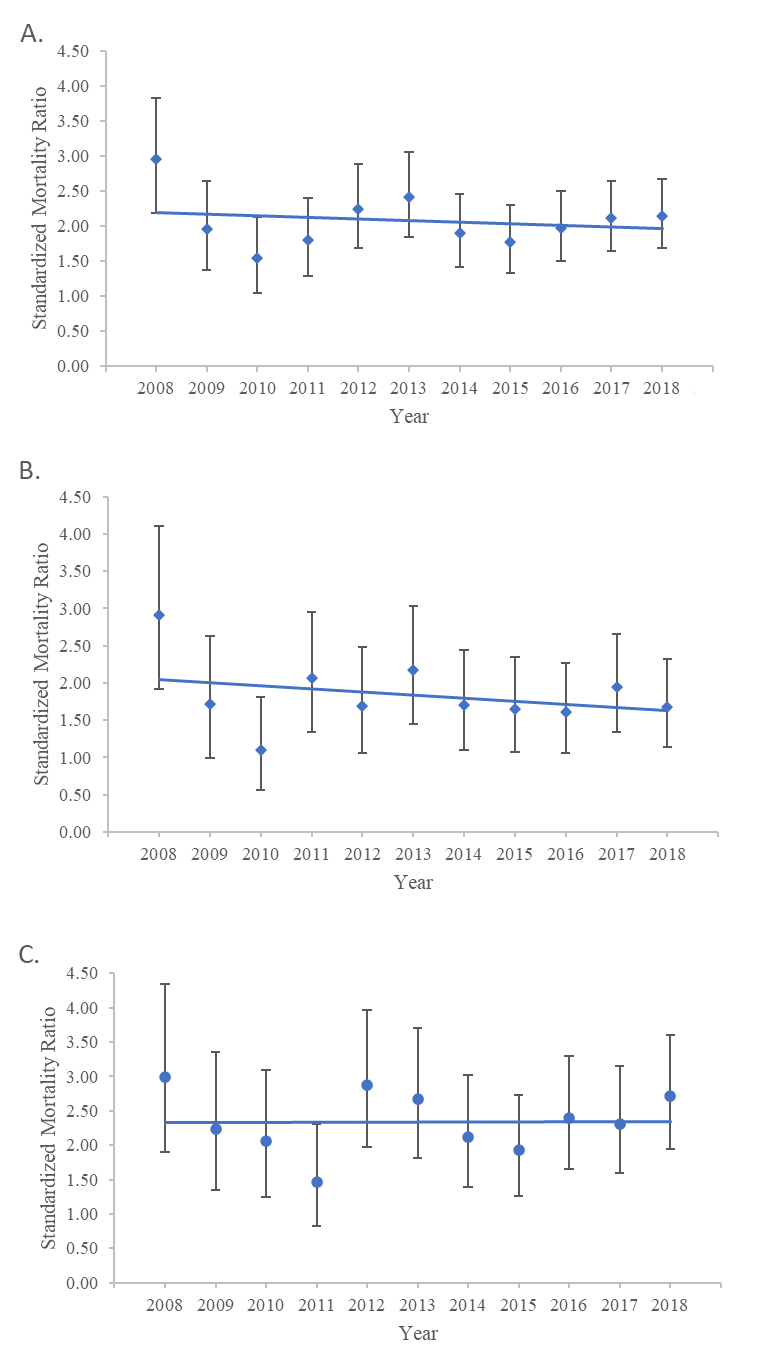


**Women**

APC = -0.14%

(95% CI: -3.91 – 4.36)

*p* = 0.940

**Men**

APC = -2.40%

(95% CI: -6.66 – 2.05)

*p* = 0.249

**Total sample**

APC = -1.00%

(95% CI: -4.46 – 2.58)

*p* = 0.538

**Fig. S1.** Annual natural-cause standardized mortality ratios of patients with bipolar disorder

over study period: (A) Total sample, (B) Men, and (C) Women

Note: APC, Annual percentage change; CI, Confidence interval.


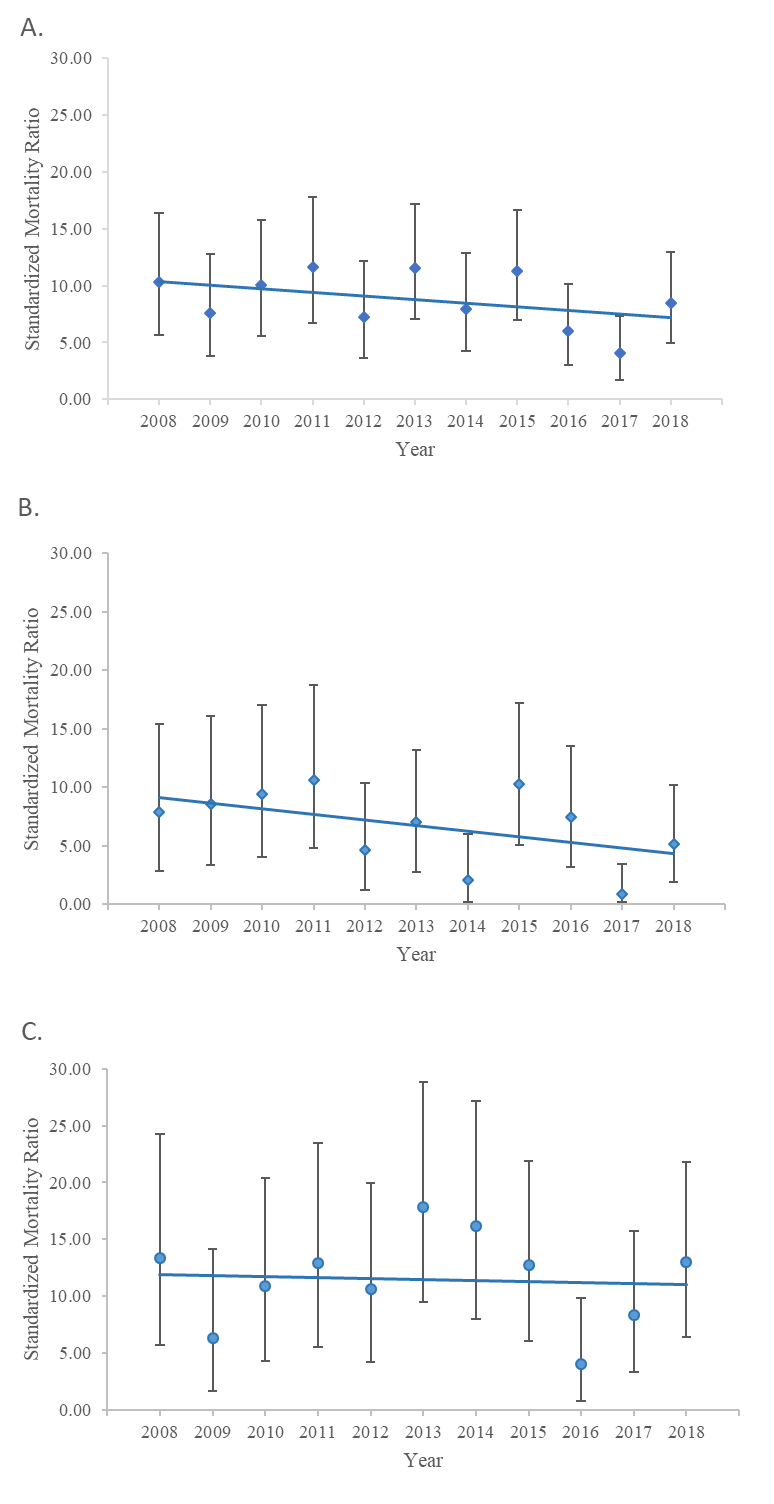


**Total sample**

APC = -3.24%

(95% CI: -9.09 – 3.00)

*p* = 0.264

**Women**

APC = -0.47%

(95% CI: -8.20 – 7.91)

*p* = 0.898

**Men**

APC = -4.57%

(95% CI: -13.43 – 5.20)

*p* = 0.306

**Fig. S2.** Annual unnatural-cause standardized mortality ratios of patients with bipolar disorder

over study period: (A) Total sample, (B) Men, and (C) Women

Note: APC, Annual percentage change; CI, Confidence interval.


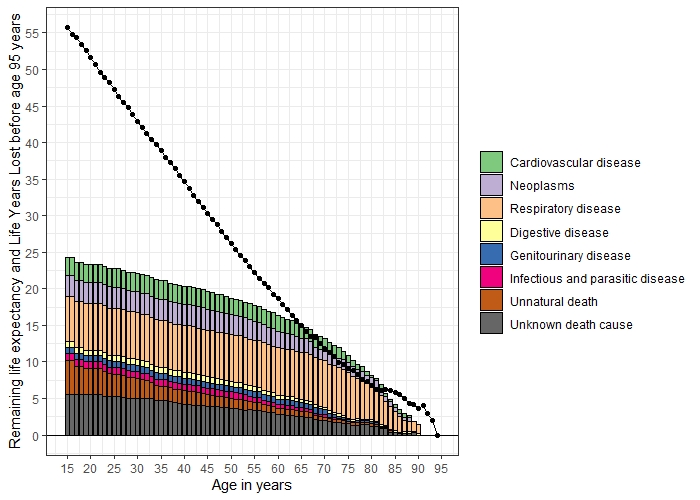

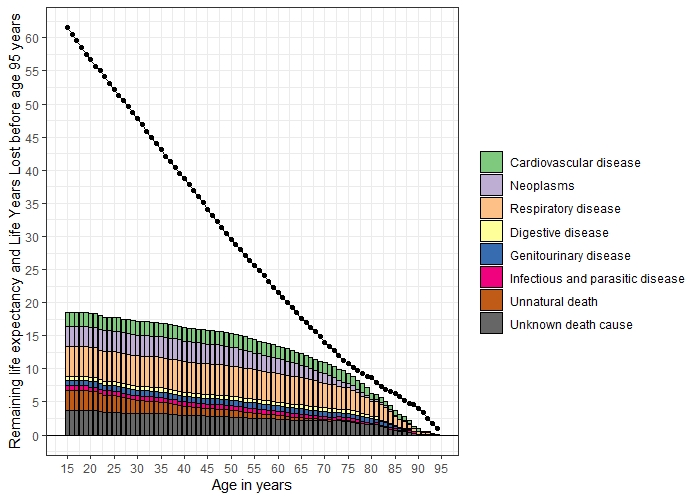


**Fig. S3.** Remaining life expectancy and cause-specific life years lost for men (left) and women (right) with bipolar disorder

The black dot indicates the remaining life expectancy (with upper-age limit set at 95 years) of patients on the y-axis at any given age of first-recorded diagnosis of bipolar disorder on the x-axis. The colored area represents the total LYLs at any given age of diagnosis on the x-axis, with each color denoting a given cause of death, and the “length” of each color area indicates the number of LYLs attributable to that specific cause of death. Respiratory diseases accounted for the largest share of LYLs for patients of both sexes, and the degree of contribution increased with age, particularly in men (proportion to total LYLs: men: 22.3% at 15 years, 60.6% at 75 years; women: 17.8% at 15 years, 38.0% at 75 years). Cancers and cardiovascular diseases also constituted a major share to LYLs due to natural-cause deaths for both men (Cancers: 5.5% at 15 years, 6.6% at 60 years; Cardiovascular diseases: 11.9% at 15 years, 16.2% at 60 years) and women (Cancers: 16% at 15 years, 19.4% at 60 years; Cardiovascular diseases: 7.0% at 15 years, 9.9% at 60 years) with bipolar disorder, with the proportion of their contributions mildly increased from 15 to 60 years. By contrast, unnatural causes accounted for a decreasing share of LYLs with age in patients of both sexes (men: 25.8% at 15 years, 7.7% at 75 years; women: 27.0% at 15 years, 3.6% at 75 years).
